# Half-Cell Potential Analysis of an Ammonia Sensor with the Electrochemical Cell Au | YSZ | Au, V_2_O_5_-WO_3_-TiO_2_

**DOI:** 10.3390/s130404760

**Published:** 2013-04-10

**Authors:** Daniela Schönauer-Kamin, Maximilian Fleischer, Ralf Moos

**Affiliations:** 1 Department of Functional Materials, University of Bayreuth, Bayreuth 95440, Germany; 2 Corporate Technology, Siemens AG, Munich 81739, Germany

**Keywords:** mixed potential, selective catalytic reduction (SCR), exhaust gas sensor, polarization curves, electrochemical cell

## Abstract

Half-cell potentials of the electrochemical cell Au, VWT | YSZ | Au are analyzed in dependence on oxygen and ammonia concentration at 550 °C. One of the gold electrodes is covered with a porous SCR catalyst, vanadia-tungstenia-titania (VWT). The cell is utilized as a potentiometric ammonia gas sensor and provides a semi-logarithmic characteristic curve with a high NH_3_ sensitivity and selectivity. The analyses of the Au | YSZ and Au, VWT | YSZ half-cells are conducted to describe the non-equilibrium behavior of the sensor device in light of mixed potential theory. Both electrode potentials provide a dependency on the NH_3_ concentration, whereby VWT, Au | YSZ shows a stronger effect which increases with increasing VWT coverage. The potential shifts in the anodic direction confirm the formation of mixed potentials at both electrodes resulting from electrochemical reactions of O_2_ and NH_3_ at the three-phase boundary. Polarization curves indicate Butler-Volmer-type kinetics. Modified polarization curves of the VWT covered electrode show an enhanced anodic reaction and an almost unaltered cathodic reaction. The NH_3_ dependency is dominated by the VWT coverage and it turns out that the catalytic properties of the VWT thick film are responsible for the electrode potential shift.

## 1. Introduction

The introduction of selective catalytic reduction (SCR) systems for exhaust gas after-treatment of NO_x_-emissions of diesel-fueled vehicles requires novel sensors for control and On-Board Diagnosis (OBD) purposes. The reducing agent ammonia, injected into the exhaust pipe as an aqueous urea solution (AdBlue^®^), reacts at the SCR-catalyst with nitrogen oxides. Nitrogen and water are formed as reaction products [1,2]. The conversion efficiency of the catalyst is, besides parameters like temperature and catalyst composition, strongly dependent on the ratio of ammonia (NH_3_) to nitrogen oxides (NO_x_), which is adjusted by the amount of AdBlue-solution injected. Selective NO_x_-sensors or NH_3_-sensors would be appropriate to monitor these concentrations downstream of the SCR-catalyst. In Reference [2–4], the control of the AdBlue dosing system by an NH_3_ sensor is preferred. It allows the closed-loop control of the SCR-system and also the OBD of the SCR-catalyst. Different NH_3_ sensing principles have been investigated for that purpose [5]. Solid electrolyte-based sensors with optimized electrode materials and configurations seem most promising. Sensors based on yttria-stabilized zirconia (YSZ) electrolyte have been studied extensively in the past years for exhaust gas applications. A robust sensor element yielding fast response, high selectivity and sensitivity, and a long-term stable sensor response is required. Besides well-known examples for high temperature applications in engine exhausts like lambda probes and amperometric NO_x_ sensors, for the detection of exhaust components like CO, H_2_, HC, or NO_x_, non-Nernstian mixed potential sensors are promising [6–14]. Mixed potential type sensors for NH_3_ detection are also under investigation by various groups [3,15,16]. Different material compositions have been screened for their applicability as sensing electrodes. For that purpose, a threefold functionality of the sensing electrode including electrical conductivity, electro-catalytic activity, and selectivity is required. Besides, long-term stability and adjusted catalytic properties are necessary. For a sufficient sensor performance, including high sensitivity, selectivity, and reliability, the sensing electrode materials need to be optimized. Very often complex material compositions, e.g., semiconducting metal oxide mixtures with additional dopants for stabilization purposes are suggested [16].

An interesting novel concept to functionalize the sensing electrodes of mixed potential type NH_3_ gas sensors is suggested in Reference [17]. It is based on the separation of the sensing electrode functionalities: two equal gold electrodes provide electrical conductivity and a three-phase boundary (TPB), whereas a separate catalyst layer on top is responsible for activity and selectivity. As catalyst layer material, vanadia-doped tungstenia titania (V_2_O_5_-WO_3_-TiO_2_, abbreviated hereafter as VWT), a commercially available SCR-catalyst for NO_x_ reduction with a proven long-term stability in the exhaust and well-known catalytic properties [18,19] is utilized. SCR active ZSM5-zeolites are also investigated as electrode coatings for this sensor concept [20,21], but a more pronounced and stable ammonia sensor signal with high ammonia sensitivity and low NO_x_ cross-interfering effects was observed for the VWT coating. The sensor voltage of this potentiometric NH_3_ sensor depends logarithmically on the NH_3_ concentration, a typical behavior for mixed-potential type sensors [22,23]. The here-discussed VWT-based sensor can be described as an electrochemical cell “VWT, Au | YSZ | Au”. It is operated at 550 °C and shows a high NH_3_-sensitivity (88 mV/decade NH_3_) with a marginal NO_x_-cross-sensitivity [17]. In more detail, the ammonia sensor response is independent on the NO concentration, even at low NH_3_ concentrations, but a small (nevertheless non-negligible) NO_2_ cross-interference is observed. It has to be considered especially at low NH_3_ concentrations. Lambda variations, *i.e.*, simultaneous variations of the concentrations of O_2_, CO_2_, and water in dependence on the combustion process in lean, oxygen rich exhaust almost do not affect the sensor response once the O_2_ concentration exceeds 3 vol.%.

This planar sensor was manufactured in thick-film technique, with a screen-printed YSZ film on top of an alumina substrate [17]. The device could be self-operated due to a heater film on its reverse side. In this study, the focus is on experiments to elucidate the sensor mechanism. For that purpose, effects contributing to the sensor response are investigated in a half-cell setup. The electrode processes at the pure Au electrode and at the VWT-covered Au electrode are analyzed separately with reference to a Pt electrode in a defined reference atmosphere. Initial investigations of the electrode effects confirmed that electrochemical reactions occurring at the three phase boundary and the catalytic activity of the VWT-catalyst layer play a crucial role in the sensor mechanism [24]. A detailed investigation of the electrode potential and electrochemical kinetics with respect to mixed potential formation is the focus of this work. Half-cell measurements of the electrode potentials are conducted in dependence on the gas composition. Polarization curves are investigated in different gas atmospheres for a Au electrode and a VWT-covered Au electrode.

The objective of this study is to validate if the sensor behavior and NH_3_ detection mechanism can be described solely by classic mixed potential theory or if other potential changing processes need to be considered which reflect limitations of the mixed potential theory.

## 2. Experimental Section

### 2.1. Sample Preparation

**Figure 1.**
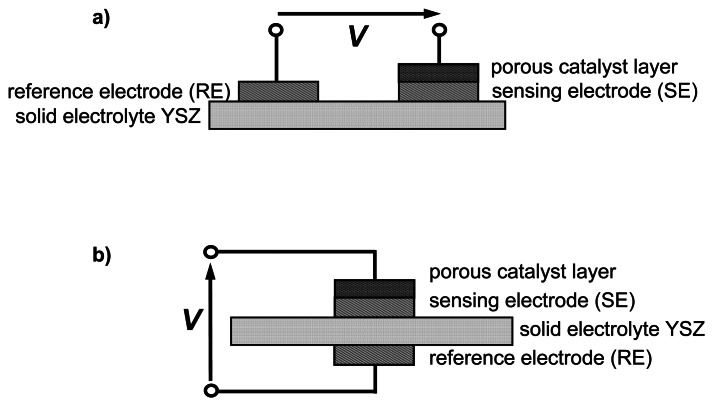
Schematic setup of the device. (**a**) Sensor-like setup. (**b**) Half-cell type setup.

Two types of samples were prepared, as illustrated in Figure 1: a sensor-like setup and a half-cell type setup. In each setup, YSZ ceramic discs (8 mol.% Y_2_O_3_-doped ZrO_2_ (Kerafol)) served as a substrate. Electrodes and cover layer were applied by screen printing. For the sensor-like setup (Figure 1(a)) both porous Au electrodes (Au thick-film paste (DuPont), fired for 20 min at 850 °C) were applied on top of the YSZ disc. One electrode, denoted as sensing electrode and abbreviated by SE, was covered afterwards by a screen-printed porous catalyst film. For that purpose, a commercial extruded VWT-based SCR catalyst (Argillon, now Johnson-Matthey) was ground in a planetary ball-mill and organic binders were added to obtain a screen-printable paste. In the case of the half-cell probe, the electrodes were arranged on top and on bottom of the electrolyte disc (Figure 1(b)). For the reference electrode (RE), which was later exposed to a reference atmosphere, either Au (processed as described) or Pt (thick-film paste (Heraeus) fired at 1,200 °C for 20 min) was used. The SE consisted of a Au layer covered by the porous SCR-catalyst VWT thick film. Different sensing electrode configurations were investigated. Half-cell probes with Au and Au that was covered partially or completely with the VWT-catalyst (applied by brushing) were used.

**Figure 2.**
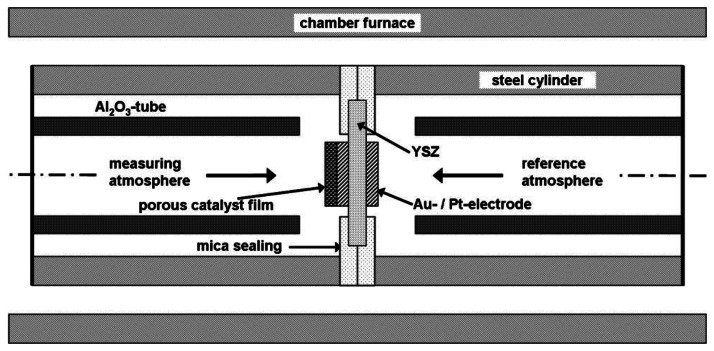
Schematic setup for half-cell measurements with half-cell probe “measuring gas, sensing electrode (SE), VWT, Au | YSZ | Au, reference electrode (RE), reference gas”.

The setup for half-cell measurements is illustrated in Figure 2. Main parts of the half-cell setup were two stainless steel cylinders, each with a gas inlet and outlet and joined together by a mica seal. Both gas atmospheres were separated gas tightly by the mica seal and the half-cell probe itself, so that both electrodes could be exposed to different gas compositions. The RE was in contact with the reference gas atmosphere whereas the SE was exposed to the measuring gas. Platinum wires were used as contact leads. The entire half-cell setup was mounted into a chamber furnace which was heated up to operation temperature. The temperature at the half-cell probe was monitored and adjusted to 550 °C by two thermocouples (not shown in Figure 2). A temperature gradient of 5 K at the maximum was observed across the half-cell specimens.

### 2.2. Measurement of Sensor and Half-Cell Performances

The sensor-like device of Figure 1(a) was measured in a tube furnace and the entire half-cell setup (Figure 2) was placed in a chamber furnace.

The sensor-like devices were mounted in a sample holder and heated up in a tube furnace to an operating temperature of 550 °C. They were exposed to a base gas consisting of 10 vol.% O_2_, 6.5 vol.% CO_2_, 2.5 vol.% H_2_O in N_2_ balance with a total flow rate of 600 mL/min. The NH_3_ concentration was increased stepwise up to 275 ppm. The sensor signal, *V*, *i.e.*, the potential difference between SE and RE as indicated in Figure 1, was recorded by a digital multimeter (Keithley 2700 series). The resulting sensor response, Δ*V*, is the difference between the sensor signal in base gas, *V*_0_, and the sensor signal with NH_3_, *V*_NH3_. The gas composition, especially the NH_3_ concentration, was analyzed by Fourier transform infrared spectrometry (FTIR, Thermo Fisher Nicolet) downstream of the tube furnace.

For half-cell tests, separate reference and measuring atmospheres were necessary. Both consisted, unless otherwise specified, of 10 vol.% O_2_, 6.5 vol.% CO_2_, and 2.5 vol.% H_2_O in N_2_ balance. The RE was always exposed to this gas composition, which was dosed constantly during the measurements. Varying NH_3_-concentrations (10–275 ppm, see Figure 5; respectively 44–470 ppm, see Figures 7, 8 and 10, 11 and 12) were added on the SE side. Additionally, it was investigated how the electrode behavior depends on NO and NO_2_ as well as on O_2_. For that purpose, NO or NO_2_ were added to the base gas or the oxygen concentration of the measuring gas was varied from 0.5 to 20 vol.%. All half-cell measurements were conducted at 550 °C.

Current-voltage curves or polarization curves were taken by a potentiostat (Autolab PGSTAT12) in a two-electrode-setup. Potential steps from −125 mV to +100 mV (steps of 25 mV, each 300 s) were applied between SE and RE and the resulting current was recorded.

**Figure 3.**
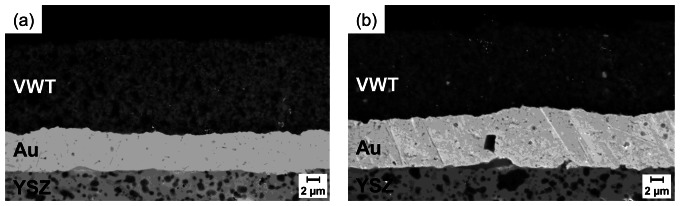
SEM-cross sections of (**a**) the sensor-like setup and (**b**) the half-cell probe.

Sample morphologies and layer thicknesses were analyzed by a scanning electron microscope LEO1450 VP (Zeiss). Figure 3 shows SEM images of cross sections of the sensor-like setup (Figure 3(a)) and the half-cell probe (Figure 3(b)). The gold electrodes have thicknesses of about 6 μm (sensor-like setup) and 8 μm (half-cell probe). Morphology and porosity of both Au electrodes are almost equal. The low roughness of the YSZ substrate can also be seen. According to [25,26] this leads to a small interface between YSZ and Au electrode. The VWT catalyst layers on top of the Au electrodes show a thickness of about 13 to 15 μm and the grain size is less than 1 μm. The images confirm the porosity of the catalyst film. The catalyst properties of both samples appear almost equal.

## 3. Results and Discussion

### 3.1. Sensor Response towards Ammonia

**Figure 4.**
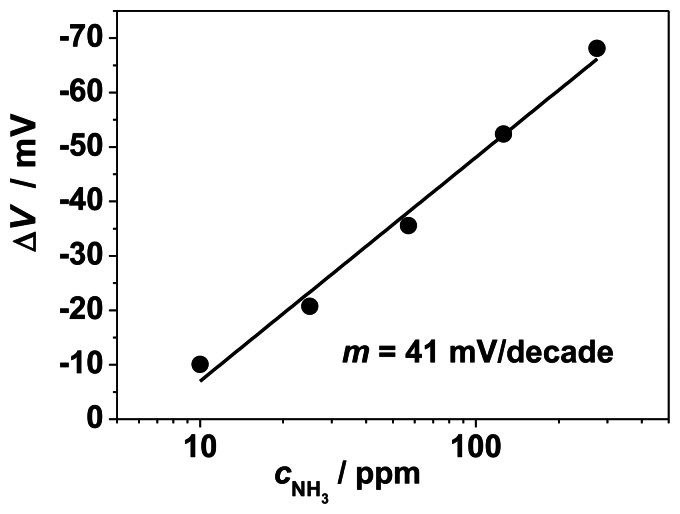
Response of the sensor-like device as shown in Figure 1(a) towards NH_3_ at 550 °C.

The characteristic curve of the sensor-like device of Figure 1(a) is shown in a semi-logarithmic representation in Figure 4. In our initial study in Reference [17], a stable sensor signal with a response time of at least 3 s and a reproducible behavior is reported. The linearity between sensor signal Δ*V* and log *c*_NH3_ is in agreement with earlier results on the planar sensor device [17] and with the mixed potential theory (e.g., [22,23,27]). The NH_3_ sensitivity at 550 °C, specified as the slope *m* of the characteristic curve, reaches 41 mV/decade *c*_NH3_. If one excluded the 10 ppm ammonia point from the fit, since it does not lay fully on the straight line, one would obtain 45 mV/decade *c*_NH3_. In Reference [17], a sensitivity of 88 mV/decade NH_3_ was found. The smaller slope may be ascribed to different electrode morphologies and differences in the three-phase boundary area and the resulting number of electrochemically active sites. The planar sensor device in Reference [17] was based on an alumina substrate equipped with a screen-printed YSZ thick film as solid electrolyte. On top of the electrolyte layer, the two gold electrodes were applied, one covered with a porous VWT thick film. As consequence, a rough surface of the YSZ thick film combined with porous electrodes was obtained. In contrast, for the here-investigated sensor-like devices, the YSZ discs provide a smooth surface with little roughness. One would expect that the sensor signal (= difference of the electrode potentials) is independent on the TPB-length. By enlarging the TPB-length all electrochemical reactions at the TPB are affected similarly, due to an increase of the number of active triple-points for each reaction. As a result, the sensitivity should be independent on the three-phase boundary length. However for the here-presented NH_3_-sensor, a dependency of the NH_3_-sensitivity was observed. It increases with an increase in surface roughness and an increasing porosity of the electrodes. This result is in accordance to Liang *et al.* [25]. Therein, the influence of the surface roughness of YSZ-substrates on the sensitivity of an NO_2_-sensor was investigated. As a result, the sensitivity increased due to an increase in surface roughness. The effect is attributed to an enhanced effective three-phase boundary area: a larger contact area is related to more electrochemically active sites for electrochemical reactions yielding an enhanced sensitivity [25].

Hence, it is assumed for the investigated NH_3_-sensor that the higher sensitivity of sensors equipped with rough YSZ-thick films and porous electrodes can be explained by the larger TPB area. In contrast, sensors with YSZ discs show a lower sensitivity due to their smoother surface and the resulting smaller TPB area. Additionally, the electrodes on top of the smooth YSZ discs provide smaller porosity. However, the surface properties of the YSZ substrates are more stable and reproducible compared to screen printed YSZ thick-films, where some scattering of the sensitivities was always observed. Therefore, the half-cell measurements were conducted with samples based on YSZ substrates.

### 3.2. Electrode Potentials

**Figure 5.**
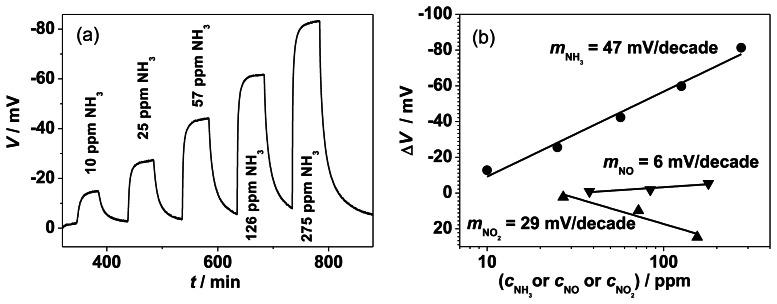
Electrode potential of the Au sensing electrode, fully covered with VWT, in dependence on the NH_3_-concentration measured with reference to a pure Au reference electrode in the half-cell setup at 550 °C. (**a**) Electrode potential *V*. (**b**) The resulting semi-logarithmic characteristic of Δ*V* = *V*_NH3_ − *V*_0_ in dependence on the NH_3_, NO and NO_2_ concentration. Please note: due to the definition that follows the standards in electrochemistry, Δ*V* and *V* have a negative sign for oxidation reactions of NH_3_ and NO and a positive sign for the reduction of NO_2_. For the sake of clarification, they are plotted reversely.

In Figure 5(a), the time dependent behavior of a half-cell measurement is shown for the half-cell probe RE, Au | YSZ | Au, VWT, SE. The electrode potential, *V*, of the fully VWT-covered sensing electrode increases when ammonia is admixed stepwise to the SE side from 10 to 275 ppm NH_3_ to the base gas of 10 vol.% O_2_, 6.5 vol.% CO_2_, 2.5 vol.% H_2_O and N_2_. Without NH_3_, if both electrodes, Au and VWT-covered Au, are exposed to the same base gas composition, a potential *V*_0_ in the range of 0 to 5 mV occurred. For other electrode materials, e.g., Au, VWT *vs.* Pt, offset voltages from 0 to 10 mV were observed. Up to now, we have only an assumption to explain the offset voltages. They may originate from the Seebeck coefficient of YSZ, which under these conditions is approximately 500 μV/K [28,29]. Hence, a small temperature difference between both electrodes of only a few K (for 5 K: 2.5 mV) might lead to such an additional voltage. Additionally, a small thermo-voltage (range of mV) could originate from the Seebeck coefficient difference of the platinum contact leads of the half-cell setup and the Au-contact wire of the used Au- and Au, VWT-electrodes of the half-cell probe. This may lead to an additional thermo-voltage, however below 1 mV.

A strong, stable, and reversible increase of the potential difference occurs when ammonia is present in the base gas (all measurements with the same sample were repeated three times; further investigations of the sensor itself (not published) showed no hysteresis effects between increasing and decreasing concentration steps; the sensitivity of one sample is stable and reproducible over at least 10 measurement cycles). This is the first indication that the electrode potential of the VWT-covered Au-electrode is responsible for the sensor effect. In contrast to the planar sensor in the exhaust [17], the response time determined by the half-cell experiments is very low since the slow gas exchange of the test bench limits the kinetics. The recovery time is apparently even lower. This is also attributed to the kinetics of the gas test bench in conjunction with the semi-logarithmic sensor characteristic (a low remaining ammonia concentration results in a marked sensor signal).

In order to quantify the NH_3_ sensitivity, the steady state values, Δ*V* = *V*_NH3_ − *V*_0_, are plotted *versus* the logarithm of the NH_3_ concentration (Figure 5(b)). Please note: due to the definition that follows the standards in electrochemistry, both Δ*V* and *V* have a negative sign for oxidation reactions of NH_3_ and NO and a positive sign for the reduction of NO_2_. In order to be coherent with the point of view of sensor developers, they are plotted reversely. In the following we will use the expression “increase” meaning the absolute value of *V* and Δ*V*.

The slope in this semi-logarithmic representation of the electrode potential Δ*V* reaches 47 mV per decade ammonia, which is in good agreement with the sensor signal discussed in the previous section (41 mV/decade *c*_NH3_). This little difference compared with the sensor-like sample is a further hint that the ammonia sensor signal can be ascribed to NH_3_-dependent changes of the electrode potential of the VWT-covered Au electrode. NO and NO_2_ show a comparable behavior obeying a semi-logarithmic law. The sensitivity towards NO is almost marginal (6 mV/decade NO), whereas the effect of NO_2_ is in opposite direction with a slope of 29 mV/decade NO_2_. In comparison to NH_3_, the NO_2_ effect is less pronounced. This agrees with results obtained for the planar sensing device published in Reference [17]. The opposite changes of the electrode potential during exposure to NH_3_ and NO_2_ can be explained by the standard electrode potentials of the assumed electrochemical electrode reactions. For the electrochemical oxidation electrode reactions (NH_3_ or NO oxidation with oxygen ions from YSZ), a negative electrode potential occurs with reference to the oxygen electrode reaction (defined at 0 V for 1 bar). For the electrochemical oxidation of NH_3_, a standard electrode potential of −1.18 V (calculated at 623 °C) is stated in Reference [38]. In case of reduction reactions like the reduction of NO_2_ (reduction of NO_2_ to NO and oxygen ions) the electrode potential is shifted to positive values. The standard electrode potential of NO_2_ reduction can be determined from [30] and is in the range of +0.05 V. The measured electrode potential is shifted from the oxygen electrode potential (defined at 0 mV) to more negative (respectively positive) electrode potentials by adding NH_3_ (respectively NO_2_) to the surrounding gas. The same overall behavior with lower sensitivities can be expected for the pure Au-electrode [14].

**Figure 6.**
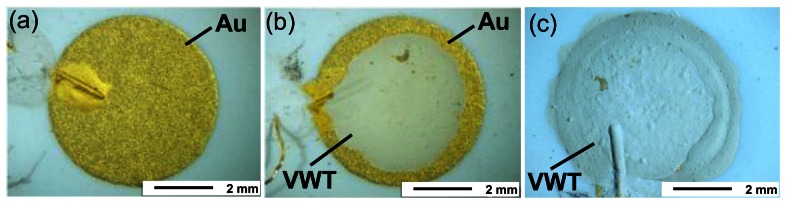
Light optical microscope images of the sensing electrode of one half-cell sample with different coverage degrees of the Au electrode with the VWT layer. (**a**) Coverage degree 0%, (**b**) coverage degree 60%, (**c**) coverage degree 100%.

To investigate the effect of the VWT thick film on the electrochemical activity more in detail, the coverage degree of the Au electrode with the VWT catalyst layer was varied. As a first experiment, one half-cell probe Au | YSZ | Au was measured to analyze the electrochemical activity of the pure Au electrode. The same specimen was afterwards partially covered with the VWT catalyst layer. After the electrode potential dependence on the ammonia concentration was determined, the electrode was completely covered with VWT. Again, the electrode potentials were determined on the same half-cell probe. The VWT-coverage degree of the Au electrode can be obtained from the light optical microscope images in Figure 6. Figure 6(a) shows the uncoated pure Au electrode, Figure 6(b) the partially VWT-coated electrode, and Figure 6(c) shows the completely coated one. Here, Pt was used as the material for the RE on the opposite side.

**Figure 7.**
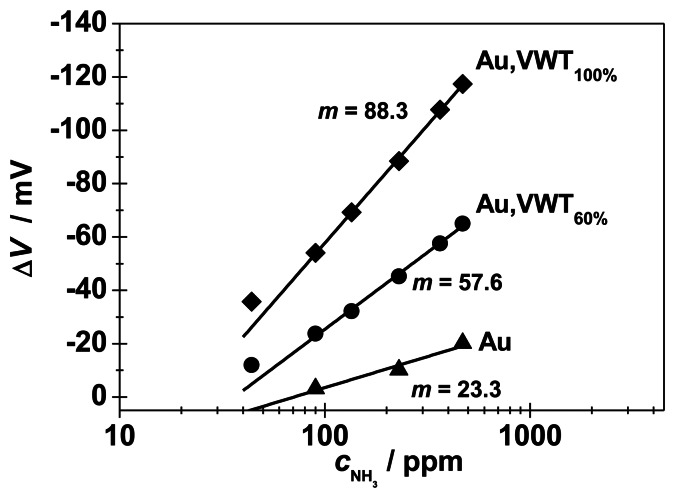
Electrode potentials Δ*V* of the half-cells Au | YSZ and Au, VWT |YSZ *versus c*_NH3_ at 550 °C. Plotted are the steady state values *versus* the logarithm of the ammonia concentration for varying VWT-coverage degrees of the same Au electrode.

Figure 7 shows the electrode potential in dependence on the NH_3_ concentration (between 44 and 470 ppm) for these three SE configurations. All yield a typical semi-logarithmic characteristic behavior with stable and reversible signals. An NH_3_-dependent electrode potential occurs for the pure gold electrode with a (small) slope of 23.3 mV/decade NH_3_. Obviously, the electrochemical activity of Au cannot be neglected, a finding which is also consistent with literature for the YSZ | Au | NO_2_ half-cell [14].

In base gas atmosphere, with 0 ppm NH_3_, the effect of the VWT-coverage can be neglected. At a VWT coverage degree of 60%, a sensitivity of 57 mV/decade NH_3_ is obtained, whereas with 100% coverage, 88 mV/decade NH_3_ are reached. The NH_3_-sensitivity *m* increases clearly with the VWT coverage degree of the Au-electrode.

**Figure 8.**
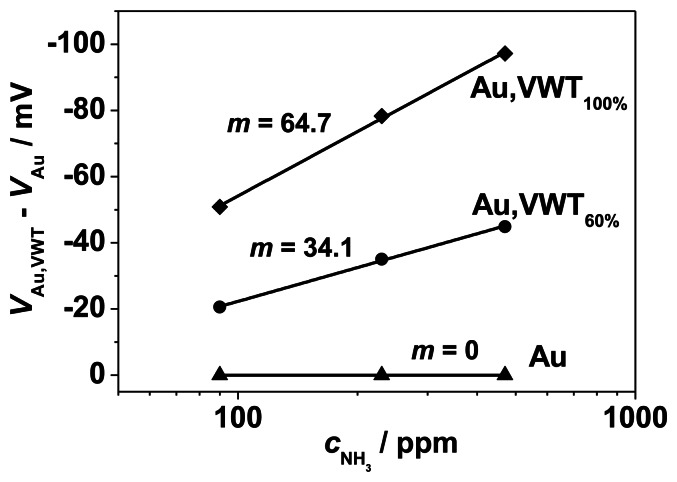
Corresponding sensor signal of the half-cell electrode potentials *V*_Au-VWT_ corrected by the NH_3_ dependency of *V*_Au_.

In the planar sensor device, both electrodes (Au and VWT-covered Au) are exposed to the analyte gas. The sensor signal corresponds to the potential difference between the Au-covered VWT electrode and the Au electrode. From half-cell investigations, the ammonia dependency of the electrode potential of the pure Au-electrode and the VWT-covered Au electrode was determined. The difference between these electrode potentials, *V*_Au,VWT_(*c*_NH3_)-*V*_Au_(*c*_NH3_), for each ammonia concentration is plotted in Figure 8. Compared to the half-cell results, the sensor signal is lower as a consequence of the electrochemical NH_3_-activity of the half-cell Au | YSZ. The results confirm the dependency of the electrode potentials on *c*_NH3_ and the higher electrochemical activity of the VWT-covered electrode.

The comparison of the slopes in Figure 4 (41 mV/decade for the sensor-like sample with both electrodes in measuring gas) and in Figure 5 (47 mV/decade for the half-cell signal of the Au, VWT-electrode with reference to a Pt electrode in reference gas) shows a difference of 6 mV/decade. It is expected that the difference is due to the contribution of the pure Au-electrode (in measuring gas). As described in [14], the electrode potential of a Au-electrode depends on the gas composition and can be correlated to the NO and NO_2_-concentration. The same behavior is assumed in case of NH_3_. Hence, a sensitivity in the range of 6 mV/decade NH_3_ was deduced for the pure Au-electrode.

As shown in the half-cell experiments in Figure 7, a slope of 23 mV/decade NH_3_ for the pure Au-electrode occurs. According to this result, the difference between the slopes of Figures 4 and 5 should be in the range of 20 mV/decade, but only 6 mV/decade have been observed.

In addition, when comparing the slopes of two half-cell measurements of the VWT-covered Au-electrode with different half-cell probes (47 mV/decade (Figure 5) and 88 mV/decade (Figure 7 Au, VWT_100%_)) a huge discrepancy is noticeable. These differences are a further hint of a non-Nernstian sensor behavior, because in case of equilibrium sensors the slopes should be equal for each sample. Here, it has to been noted that the experiments are tested with different samples and as known for mixed-potential sensor devices the scattering from sample to sample is a serious issue. As a consequence with respect to equal test conditions, the difference in the slopes can only be ascribed to fluctuations between actually similar samples (like differences in morphology, electrode and catalyst thicknesses, and interfaces YSZ | electrodes).

According to mixed potential theory, two electrochemical reactions compete at the TPB and form a mixed electrode potential [22,23,31,32]. A mixed potential establishes if the reaction rates of the anodic and the cathodic electrochemical reaction are equal, *i.e.*, the total net current flux equals zero. The mixed potential depends therefore on the reaction rates of the proceeding electrochemical reactions. In case of an ammonia sensor and an oxygen ion conducting electrolyte, the electrochemical oxidation of ammonia (1) and the reduction of oxygen (2) at the TPB are assumed to be relevant reactions. The reaction equations are written in Kröger-Vink defect notation [33]: V_O_^••^ denotes a doubly positively charged oxygen vacancy in the solid electrolyte YSZ, whereas O_O_^x^ stands for electrically neutral oxygen ions on oxygen lattice sites of YSZ and e ′ represents a conduction electron:

(1) $$\frac{2}{3}\;NH_{3}+{O_{o}}^{x}↔H_{2}O+\frac{1}{3}N_{2}+2e\prime +V_{o}^{••}$$

(2) $$\frac{1}{2}\;O_{2}+2e\prime \;+V_{o}^{••}↔{O_{o}}^{x}$$

From References [3] and [24], an expression for the mixed potential is deduced from Equations (1) and (2), leading to a theoretical slope of 54.4 mV/decade NH_3_ at 550 °C. This value represents a first approximation of the resulting mixed potential, but considers only parameters like the partial pressures of NH_3_, O_2_, and H_2_O and the temperature. The reaction rates of the electrochemical reactions, catalytic properties of the electrodes, and adsorption properties of the electrodes are not included [10,27,34–36]. More comprehensive approaches for theoretical description of the mixed potential can be found in References [10,27,32,34]. The classic mixed potential theory [10,22,34] bases on the Butler-Volmer equation, which can be investigated with *I*-*V*-curves. Another approach to explain the sensor behavior of non-Nernstian sensors is the differential electrode equilibria theory. The there-discussed sensing mechanism includes (besides the mixed potential theory) further electrode contributions like chemisorption processes and changes in the Fermi level of the electrode material, especially in case of semi-conducting oxide sensing electrodes [35–37].

Additionally, the catalytic properties of the applied electrode materials need to be considered [35–37], in our case with respect to the increasing sensitivity due to VWT coverage. Besides the well-known SCR-activity of VWT, especially in a lower temperature range, the oxidation of NH_3_ with gaseous O_2_ seems to play an important role at 550 °C. In Reference [24], conversion data of VWT powder are published, obtained in a vertical reactor at 550 °C using 1 g VWT-powder. It is shown that 50% of the ammonia are oxidized in a gas phase reaction with gaseous oxygen, leading to the well-known reaction products NO, NO_2_, N_2_O, and N_2_ [18,38].

The catalytic conversion efficiency of the VWT thick film on top of the sensor device differs from that of the VWT powder due to a difference in the residence time of ammonia. For the vertical reactor with an external gas flow of 1 L/min a gas velocity of 3.4 cm/s can be calculated. The packed bed of the VWT powder had a height of approximately 0.3 cm. A residence time *t*_VWT powder_ of the gas in the powder of around 90 ms results and a conversion of 50% NH_3_ was achieved. In contrast, the VWT layer on top of the sensing electrode of the sensor device provides a thickness of 10 to 15 μm and only a small amount of powder (some mg) is necessary for that layer. The gas flow through the catalyst thick film is governed by diffusion. The diffusion through a porous medium can be described by the effective diffusion coefficient for ammonia *D*_NH3, eff_ [39,40] and is calculated by:

(3) $$D_{NH3,e\;f\;f}=D_{NH3,\text{gas}}\cdot \frac{ɛ}{\tau }$$

with the gas diffusion coefficient of ammonia, *D*_NH3, gas_, the porosity of the catalyst layer, ε, and the tortuosity factor, τ. The parameter ε/τ is determined to be approximately 0.055 with a porosity of the VWT thick film of 30% evaluated from Figure 3(a) [40]. The calculated effective diffusion coefficient at 550 °C is *D*_NH3, eff_ = 0.067 cm^2^/s. For a diffusion length *d*_VWT, thick film_ of 10 μm (= VWT layer thickness) a residence time *t*_VWT thick film_ of 7 μs can be estimated:

(4) $$t_{\text{VWT},\text{thickfilm}}=\frac{{d_{\text{VWT},\text{thickfilm}}}^{2}}{2\cdot D_{NH3,e\;f\;f}}$$

Obviously, NH_3_ resides in the fixed bed by far longer in the VWT catalyst than in the case of the thick film electrode cover. Hence, one can expect that the ammonia oxidation on the VWT thick film is by far lower than the 50% achieved in the fixed bed reactor. As a consequence of the low conversion efficiency most of the ammonia diffuses through the VWT cover layer without being oxidized. Only a fraction of ammonia is oxidized to NO_x_, N_2_, and N_2_O. Besides ammonia the reaction products, mainly N_2_ and NO_x_, reach the TPB.

The gas composition present at the TPB differs from that in the gas phase due to the catalytic conversion at VWT. The concentration of NH_3_ is only slightly lower than in the gas phase. The small oxygen concentration change (change in the ppm range, compared to an entire *c*_O2_ of 10 vol.% in the gas phase) is considered as marginal. Additionally, the NO and NO_2_ concentration at the TPB are higher (some ppm) compared to the gas phase (0 ppm). It is suggested that, in sum, O_2_, NH_3_, NO and NO_2_ are present at the TPB and each of these gas components could contribute by an electrode reaction to the measured electrode potentials. However, by comparing the marginal NO sensitivity and the opposite NO_2_ signal to the strong NH_3_ response, it is deduced that the contribution of the reaction products NO and NO_2_ to the electrode potential play only a minor role. The electrode reaction of ammonia seems to explain the electrode potential shift. The effects of NO, NO_2_, and NH_3_ on polarization curves will be shown and discussed in Section 3.3 (see Figure 13).

It is assumed that both electrochemical reactions ((1) and (2)) occur simultaneously at the TPB Au | YSZ | O_2_, NH_3_ yielding to the NH_3_ dependence of the electrode potential *V*_Au_. For the pure Au electrode, all NH_3_ can reach the TPB, but only a small fraction of the gaseous NH_3_ is oxidized electrochemically due to the low electrochemical NH_3_ activity of Au (low sensitivity). As discussed previously, most of the NH_3_ reaches the TPB, even if the Au electrode is covered with VWT and then both electrochemical reactions can proceed. With respect to mixed potential theory, the reaction rates of the concurring electrochemical reactions (1) and (2) determine the formed mixed potential. It is assumed that the VWT catalyst layer changes either the oxygen reduction reaction or/and alters the ammonia oxidation reaction. According to SCR theory the initial step of the SCR mechanism is the adsorption of ammonia on vanadia sites followed by an activation of NH_3_ [41]. The activated NH_3_ species could be responsible for the increased electrochemical activity. In [42], a similar behavior was observed. It has to be mentioned that in contrast to the here discussed ammonia sensor setup, the influence of the catalytic activity of the sensing electrode material itself, directly related to the TPB, was investigated in [42]. The sensitivity of the presented CO sensor increases with increasing catalytic activity of the sensing electrode material due to changes in the anodic and cathodic reactions.

**Figure 9.**
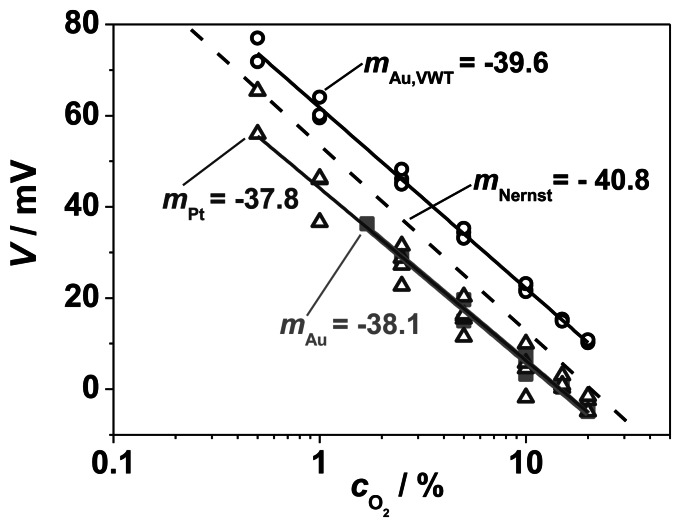
Electrode potentials, *V*, of the half-cells Au | YSZ, Pt | YSZ and Au, VWT | YSZ *versus* oxygen concentration, *c*_O2_, at 550 °C. Plotted are the steady state values *versus* the logarithm of the oxygen concentration for varying electrode materials (Au, Pt and VWT-covered Au) and the calculated Nernstian response (dashed line).

In order to obtain more information about the electrochemical oxygen reaction, the dependency of the electrode potential on the O_2_ concentration was investigated with the half-cell experiments. For that purpose, the oxygen concentration on the measuring side was varied stepwise from *c*_O2_ = 0.5 vol.% to *c*_O2_ = 20 vol.%, whereas a constant concentration of *c*_O2_ ≈ 21 vol.% (compressed air) flew on the reference side. Here, only *c*_O2_ was changed and no NH_3_ was added. The steady state values of the measured electrode potentials for three different electrode configurations (pure Au, pure Pt, and completely VWT-covered Au, measured against a Pt RE) are plotted *versus* the oxygen concentration in Figure 9. The straight dashed line indicates the expected Nernstian response. From the Nernst equation, a theoretical slope of −40.8 mV/decade O_2_ is calculated at 550 °C. The curves of the electrode potentials of Pt, Au and Au, VWT in dependence on *c*_O2_ confirm the Nernstian behavior for each investigated electrode material, since the resulting slopes (−38 to −39 mV/decade O_2_) agree with the calculated Nernst response at 550 °C. The electrodes behave as expected for an equilibrium process. The electrochemical oxygen reactions are not affected by the VWT catalyst coating. The same behavior was found for pure Pt, pure Au, and VWT-covered Au. The voltage shift of the Au, VWT electrode to higher values corresponds to the measured offset voltage when both electrodes (SE and RE) are exposed to the same oxygen concentration (20 *vs.* 21 vol.% O_2_). An offset voltage of 10 mV was determined for the Au, VWT electrode whereas a smaller offset (0 to −4 mV) was observed for Au and Pt. As explained before, the offset voltage could be explained by a temperature gradient over the half-cell probe resulting in a thermo-voltage due to the high Seebeck coefficient of YSZ [29]. A further small contribution (<1 mV) may stem from thermo-voltages of the lead materials. In summary, the electrodes provide a comparable electrochemical oxygen activity, independent on the VWT coverage.

In addition, the results in Figure 9 demonstrate the tightness of the half-cell setup. Even at a high oxygen concentration difference between reference and measuring side (21 vol.% compared to 0.5 vol.% O_2_), the Nernstian behavior indicates a negligible gas exchange between both gas compartments.

It is concluded from the Nernstian behavior that the electrochemical gaseous oxygen reaction on the TPB boundary according to Equation (2) is not affected by the VWT catalyst layer and that the fully VWT-covered electrode behaves also like an equilibrium oxygen electrode. But it has not yet been proven how the reaction rates of the oxygen reduction, and especially of the ammonia oxidation, behave if ammonia is present in the gas atmosphere. Therefore, voltage-current or polarization curves were conducted according to the classic mixed potential theory [10,22,34] based on the Butler-Volmer equation.

### 3.3. Polarization Curves

**Figure 10.**
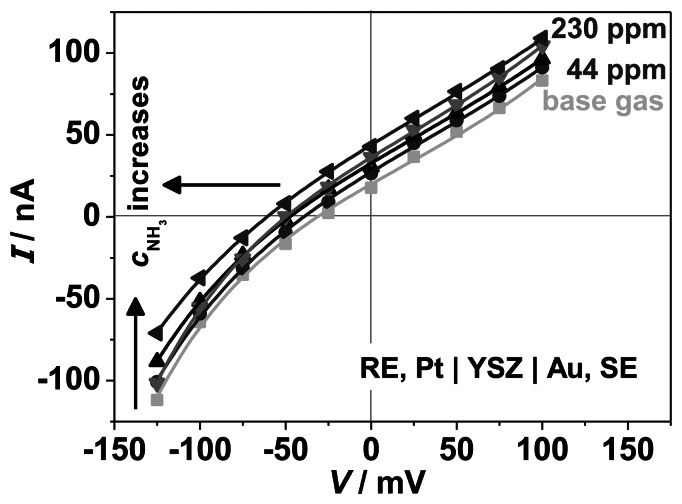
Polarization curves for the half-cell Au | YSZ with reference to a Pt electrode, recorded at 550 °C, as it depends on the ammonia concentration, *c*_NH3_, as indicated.

**Figure 11.**
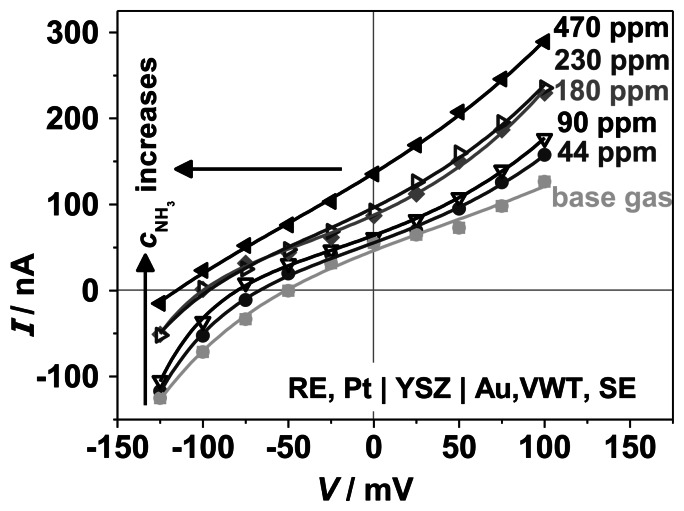
Polarization curves for the half-cell VWT, Au | YSZ with reference to a Pt electrode, recorded at 550 °C, as it depends on the ammonia concentration, *c*_NH3_, as indicated.

Polarization curves were performed to analyze the effect of the VWT catalyst coating on the electrochemical characteristics. *I*-*V*-curves were taken on the same half-cell probe RE, Pt | YSZ | Au, (VWT), SE, onto which VWT was added subsequently. The results of the polarization curves are shown in Figures 10 and 11.

A voltage from −125 to 100 mV (25 mV steps, 300 s each) was applied to the SE and the resulting current between SE and RE was measured. Figure 10 shows the NH_3_ dependency of the *I*-*V*-curves of the cell Au | YSZ. The electrode potential, *V*_0_*,* corresponding to the voltage at a current of 0 nA, shifts with increasing ammonia concentration to more negative potentials. In base gas, a *V*_0_ ≈ −25 mV can be determined, whereas with 230 ppm NH_3_ a value of − 60 mV occurs. The shape of the curves remains unaffected by the ammonia concentration. At a fixed potential, *i.e.*, in an amperometric operation mode, the current increases slightly with increasing NH_3_ concentration. It is suggested that more charge can be transferred due to generated electrons during the electrochemical NH_3_ oxidation (Equation (1)). However, one should notice here that the measured current is less than 100 nA, indicating that only a small current is generated by the electrochemical reactions.

In contrast, the VWT catalyst layer influences strongly the polarization behavior of the Au, VWT | YSZ half-cell (Figure 11) when NH_3_ is present in the gas. A strong shift of the electrode potential *V*_Au,VWT_ to negative values and a changed curve shape occurs. The shift can be explained by the standard electrode potential of NH_3_ oxidation in equilibrium. In Reference [30], a value of − 1.18 V was calculated from thermodynamic data at 623 °C related to the electrode reaction with O_2_ (0 V for 1 bar). This indicates the contribution of the electrochemical oxidation of ammonia to the electrode potential resulting in the formation of a mixed potential. The measured standard electrode potential is close to the oxygen reaction potential and therefore still dominated by the electrochemical oxygen reaction with an additional mixed potential. Interestingly, contrary to the pure Au electrode, the shape of *I*-*V*-curves changes also. These effects and the observed higher current may be attributed to the electrochemical NH_3_-oxidation at the TPB Au, VWT | YSZ, which is supported probably by the catalytic active VWT-layer. At a constant applied potential, the resulting current and the current change during ammonia addition is higher compared to the pure Au electrode. Different electrochemical kinetics of the pure Au and the VWT-covered Au electrode, due to the catalytic properties of the VWT film, could be the reason [42]. Additionally, the adsorbed species and their activity can be affected by the VWT film and influence the electrode potential [30].

**Figure 12.**
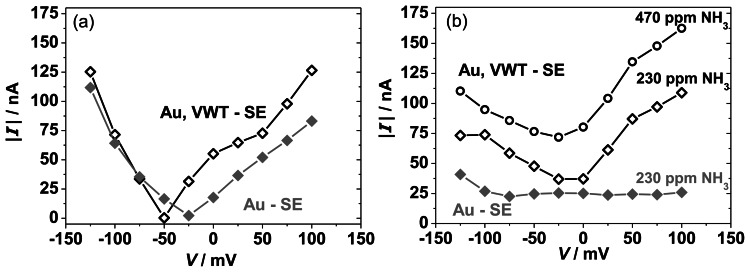
Modified polarization curves for the half-cells Au | YSZ and VWT, Au | YSZ. (**a**) Cathodic curve (O_2_ reduction) in base gas (10 vol.% O_2_, 6.5 vol.% CO_2_, 2.5 vol.% H_2_O, N_2_). (**b**) Anodic curve (NH_3_ oxidation) in 230 ppm and 440 ppm NH_3_ added to the base gas at 550 °C.

The reaction rates are characterized by the exchange current and can be analyzed by Butler-Volmer and mixed potential theory [10,34]. The polarization curves represent the kinetic behavior of the electrode and accordingly, the mixed potential is formed when the anodic and cathodic reaction proceed with an equal reaction rate. To quantify the anodic NH_3_ oxidation reaction (Equation (1)) and the cathodic O_2_ reduction reaction (Equation (2)) separately, modified polarization curves have been calculated [10,42,43] as described below. Figure 12 compares the modified polarization curves for the cathodic O_2_ reduction (Figure 12(a)) and the anodic NH_3_ oxidation (Figure 12(b)) for the half-cells Au | YSZ and VWT, Au | YSZ at 550 °C. In Figure 12(a), for the measurement in lean base gas, the absolute current values are plotted *versus* the potential, *V*. The cathodic modified polarization curves (Figure 12(a)) correspond to the absolute current values of the base gas curves in Figure 10 respectively Figure 11. For the anodic reaction, *I*-*V*-curves have been recorded in base gas atmosphere with NH_3_, so the cathodic oxygen reaction proceeds also. The anodic polarization curves (Figure 12(b)) are determined by subtracting the absolute current values in base gas (current values for base gas from Figures 10 and 11) from the current values measured in base gas with 230 ppm NH_3_ (current values for 230 ppm NH_3_ from Figures 10 and 11).

The modified polarization curves for the cathodic reaction in base gas (Figure 12(a)) for Au and VWT-covered Au electrodes behave almost similar. Only negligible differences are visible. It is assumed that the cathodic reaction is barely changed by the VWT cover. This agrees with the characteristics of the electrode potential presented in Figure 9. Again, it has to been noticed, that the current is very low (<100 nA), indicating that the reaction rate of the oxygen reduction is small and hardly proceeds at the three-phase boundaries of both half-cells. In contrast, the anodic polarization curves illustrated in Figure 12(b) behave differently. The current generated by the anodic NH_3_ oxidation on the pure Au electrode is almost constant (25 nA) over the investigated potential range. The VWT-covered Au electrode provides a higher current, but the current is still in the nA-range. The difference in the modified anodic polarization curves indicates that the VWT cover of the Au electrode enhances the anodic NH_3_ reaction, whereas the cathodic O_2_ reaction is only marginally influenced by the VWT layer. With increasing NH_3_ concentration, a higher current can be observed, *i.e.*, more ammonia can be oxidized in the anodic reaction.

**Figure 13.**
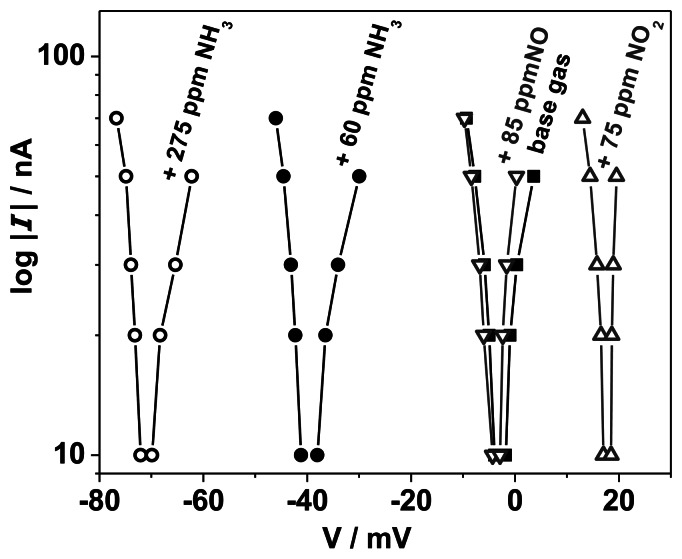
*V*-log |*I* |-curves of the half-cell Au, VWT | YSZ recorded in base gas and after addition of NO (85 ppm), NO_2_ (75 ppm), or NH_3_ (60 and 275 ppm) to the base gas at 550 °C. Au served as a reference electrode.

In addition, voltage-current curves of the half-cell Au, VWT | YSZ were recorded during exposure to NO and NO_2_ in base gas. Here, a Au-reference electrode, exposed to constant base gas was used. In this case a current (− 90 to +90 nA, 10 nA steps) was impressed to the sample and the resulting voltage was measured (instead of applying a voltage and measuring the current). The determined *V*-log |*I* |-curves are shown in Figure 13 for base gas and base gas with NO (85 ppm), NO_2_ (75 ppm), or NH_3_ (60 and 275 ppm). All curves behave as expected from Butler-Volmer theory. The electrode potential can be determined from the intersection of the curves with the abscissa at a current of *I* = 0. An electrode potential of − 3 mV occurs in base gas. It remains almost constant when NO is added (− 3.5 mV). A larger effect occurs with NO_2_. As expected, the potential is shifted to +18 mV towards the standard NO_2_ reduction potential. The largest potential change to − 40 mV (60 ppm) respectively − 71 mV (275 ppm) is observed when NH_3_ is added to the base gas. This corresponds with the expected potential change towards the standard electrode potential of NH_3_ oxidation (− 1.18 V). These results are in good agreement with the previously determined electrode potentials.

It becomes clear from Figure 13 that the kinetic behavior is dominated by the alteration of the electrode potential, whereas the shape of the curves is almost unaffected by the gas composition. This can be explained by the difference in the gas concentrations of the involved gas species. Oxygen is permanently present in excess (10 vol.%), whereas the test gases (NH_3_, NO and NO_2_) are added only in the ppm range. It can be deduced that the electrode potential is dominated by the oxygen reaction and the oxygen standard electrode potential (defined at 0 mV). At a lower O_2_ concentration (around 1 vol.%), an increased sensitivity to NH_3_ was observed [17]. In this case, the influence of the oxygen concentration on the resulting electrode potential decreases and the ammonia effect on the value of the electrode potential increases. The electrode potential of the Au, VWT | YSZ half-cell is shifted to − 220 mV at 1 vol.% O_2_ and 470 ppm NH_3_ compared to − 90 mV for 10 vol.% O_2_ and 470 ppm NH_3_. At about 0 vol.% O_2_ (no O_2_ was added to the gas feed, but a low oxygen partial pressure is still present) and 470 ppm NH_3_ an electrode potential of − 380 mV was determined (not shown here). The electrode potential shifts with decreasing oxygen concentration to more negative values towards the standard potential of ammonia oxidation. The effect of NH_3_ concentration becomes stronger with decreasing *c*_O2_, but still both electrode reactions define the electrode potential. This is a further hint for mixed potential mechanism.

A smaller effect occurs during exposure to NO_2_ and especially NO. The influence of the NO concentration on the *V*-log |*I* |-curves is marginal and the effect of the NO concentration at the TPB on the electrode potential can be neglected. From catalytic conversion data on VWT catalyst powder it is deduced that only a small NO conversion to NO_2_ occurs on the catalyst layer [44]. Additionally adsorption of NO on VWT can be neglected according to the SCR mechanism [41,45,46]. Therefore, the concentration of NO at the TPB is equivalent to that in the gas phase. As result, the half-cell Au, VWT | YSZ is almost insensitive to NO. A corresponding result for the half-cell Au | YSZ was found in Reference [14]. In contrast, NO_2_ yields to a marked change in electrode potential, a shift to positive electrode potentials occurs. At the catalyst layer, a small fraction of NO_2_ is converted to NO, but most of the NO_2_ in the gas phase also reaches the TPB. An electrode reaction of NO_2_ at the TPB is expected. It results in the shift of the electrode potential towards the standard electrode potential of NO_2_ reduction. These results are consistent with the results for the half-cell Au | YSZ in [14].

In summary, the results prove that electrochemical reactions with NH_3_ and NO_2_ take place at the TPB, whereas NO yields a marginal effect. The different shifts (direction and magnitude) of the electrode potentials due to NH_3_ or NO_2_ exposure confirm the assumption that the NH_3_, which reaches the TPB, contributes to an electrode reaction itself and the reaction products of the NH_3_ oxidation at the catalyst layer (mainly N_2_, NO and NO_2_) are not responsible for the sensor effect. Hence, a small overlapping effect of the NO_2_ electrode reaction is conceivable.

## 4. Conclusions

Half-cell measurements of Au | YSZ and Au, VWT | YSZ demonstrate that both electrode potentials depend on the ammonia concentration and are shifted in the anodic direction. The NH_3_ sensitivities agree well with sensor results presented in [17], and allow a deeper understanding of the characteristics of this sensor. The potential of the VWT-covered Au electrode, *V*_Au,VWT_, is strongly influenced by NH_3_ exposure and dominates the sensor characteristics. The potential shift confirms the formation of mixed potentials at both half-cells. The sensitivity increases clearly with increasing VWT coverage of the Au electrode, confirming that the VWT layer is responsible for the sensor effect. Additionally, the electrochemical kinetic seems to be affected by the VWT catalyst layer. Polarization curves and the calculated modified polarization curves verify Butler-Volmer behavior of the electrochemical reactions. However, the low current (range below 200 nA) is an evidence for slow electrochemical conversion. The VWT coverage enhances the anodic reaction of NH_3_, whereas the cathodic oxygen reaction remains almost unchanged, resulting in a strong shift of the electrode potential *V*_Au,VWT_ in the anodic direction. The catalytic properties of the VWT layer seem to be responsible for the enhancement of the electrochemical NH_3_ oxidation in contrast to the pure Au-electrode. Besides the formation of an activated ammonia species which could be responsible for the enhanced electrochemical activity, adsorption processes and surface effects on the electrodes could be involved, as discussed in differential electrode equilibria theory.

## References

[1] Koebel M., Elsener M., Kleemann M. (2000). Urea-SCR: A promising technique to reduce NOx emissions from automotive diesel engines. Catal.Today.

[2] Kašpar J., Fornasiero P., Hickey N. (2003). Automotive catalytic converters: Current status and some perspectives. Catal.Today.

[3] Wang D., Yao S., Shost M., Yoo J., Cabush D., Racine D., Cloudt R., Willems F. (2009). Ammonia sensor for closed-Loop SCR control. SAE Int. J. Passeng. Cars-Electron. Electr. Syst..

[4] Herman A., Wu M., Cabush D., Shost M. (2009). Model based control of SCR dosing and OBD strategies with feedback from NH_3_ sensors. SAE Int. J. Fuels Lubr..

[5] Moos R., Schönauer D. (2008). Recent developments in the field of automotive exhaust gas ammonia sensing. Sens. Lett..

[6] Moos R. (2005). A brief overview on automotive exhaust gas sensors based on electroceramics. Int. J. Appl. Ceram. Technol..

[7] Fergus J.W. (2007). Solid electrolyte based sensors for the measurement of CO and hydrocarbon gases. Sens. Actuators B Chem..

[8] Lu G., Miura N., Yamazoe N. (1996). High-temperature hydrogen sensor based on stabilized zirconia and a metal oxide electrode. Sens. Actuators B Chem..

[9] Sorita R., Kawano T. (1997). A highly selective CO sensor using LaMnO_3_ electrode-attached zirconia galvanic cell. Sens. Actuators B Chem..

[10] Miura N., Raisen T., Lu G., Yamazoe N. (1998). Highly selective CO sensor using stabilized zirconia and a couple of oxide electrodes. Sens. Actuators B Chem..

[11] Mukundan R., Brosha E.L., Brown D.R., Garzon F.H. (1999). Ceria-electrolyte-based mixed potential sensors for the detection of hydrocarbons and carbon monoxide. Electrochem. Solid State Lett..

[12] Guth U., Zosel J., Jakobs S., Westphal D., Müller R. (2002). Au–oxide composites as HC-sensitive electrode material for mixed potential gas sensors. Solid State Ionics.

[13] Miura N., Lu G., Yamazoe N. (1998). High-temperature potentiometric/amperometric NO_x_ sensors combining stabilized zirconia with mixed-metal oxide electrode. Sens. Actuators B Chem..

[14] Kubinski D.J., Visser J.H., Soltis R.E., Parsons M.H., Nietering K.E., Ejakov S.G., Kale G.M., Akbar S.A., Liu M. (2002). irconia-Based Potentiometric NO_x_Sensor Utilizing Pt and Au Electrodes. Ceramic Transactions (Chemical Sensors for Hostile Environments).

[15] Elumalai P., Plashnitsa V.V., Fujio Y., Miura N. (2008). Stabilized zirconia-based sensor attached with NiO/Au sensing electrode aiming for highly selective detection of ammonia in automobile exhausts. Electrochem. Solid State Lett..

[16] Wang D.Y., Symons W.T., Farhat R.J., Valdes C.A., Briggs E.M., Polikarpus K.K., Kupe J. (2006). Ammonia Gas Sensors.

[17] Schönauer D., Wiesner K., Fleischer M., Moos R. (2009). Selective mixed potential ammonia exhaust gas sensor. Sens. Actuators B Chem..

[18] Busca G., Lietti L., Ramis G., Berti F. (1998). Chemical and mechanistic aspects of the selective catalytic reduction of NO_x_ by ammonia over oxide catalysts: A review. Appl. Catal. B.

[19] Kröcher O., Elsener M. (2008). Chemical deactivation of V_2_O_5_/WO_3_-TiO_2_ SCR catalysts by additives and impurities from fuels, lubrication oils, and urea solution—I. Catalytic studies. Appl. Catal. B.

[20] Sahner K., Hagen G., Schönauer D., Reiβ S., Moos R. (2008). Zeolites-Versatile materials for gas sensors. Solid State Ionics.

[21] Schönauer D., Wiesner K., Fleischer M., Moos R. Einfluss der Katalysatorzusammensetzung auf das Verhalten eines mischpotentialbasierten Ammoniaksensors (in German). Dresdner Beiträge zur Sensorik.

[22] Fergus J.W. (2011). Sensing mechanism of non-equilibrium solid-electrolyte-based chemical sensors. J. Solid State Electrochem..

[23] Miura N., Elumalai P., Plashnitsa V.V., Ueda T., Wama R., Utiyama M., Comini E., Faglia G., Sberveglieri G. (2009). Solid-State Electrochemical Gas Sensing. Solid State Gas Sensing.

[24] Schönauer D., Wiesner K., Fleischer M., Moos R. (2011). Investigation of the electrode effects in mixed potential type ammonia exhaust gas sensors. Solid State Ionics.

[25] Liang X., Yang S., Li J., Zhang H., Diao Q., Zhao W., Lu G. (2011). Mixed-potential-type zirconia-based NO_2_ sensor with high-performance three-phase boundary. Sens. Actuators B Chem..

[26] Plashnitsa V.V., Elumalai P., Fujio Y., Miura N. (2009). Zirconia-based electrochemical gas sensors using nano-structured sensing materials aiming at detection of automotive exhausts. Electrochim.Acta.

[27] Guth U., Zosel J. (2004). Electrochemical solid electrolyte gas sensors—Hydrocarbon and NOx analysis in exhaust gases. Ionics.

[28] Röder-Roith U., Rettig F., Röder T., Janek J., Moos R., Sahner K. (2009). Thick-film solid electrolyte oxygen sensors using the direct ionic thermoelectric effect. Sens. Actuators B Chem..

[29] Ahlgren E., Poulsen F.W. (1994). Thermoelectric power of YSZ. Solid State Ionics.

[30] Göpel W., Reinhardt G., Rösch M. (2000). Trends in the development of solid state amperometric and potentiometric high temperature sensors. Solid State Ionics.

[31] Brosha E.L., Mukundan R., Brown D.R., Garzon F.H., Visser J.H. (2002). Development of ceramic mixed potential sensors for automotive applications. Solid State Ionics.

[32] Pijolat C., Viricelle J., Fleischer M., Lehmann M. (2012). Development of Planar Potentiometric Gas Sensors for Automotive Exhaust Application. Solid State Gas Sensors—Industrial Application, Springer Series on Chemical Sensors and Biosensors.

[33] Kröger F.A., Vink H.J., Seitz F., Turnbull D. (1956). Relations between the concentrations of imperfections in crystalline solids. Solid State Physics.

[34] Park C.O., Fergus J.W., Miura N., Park J., Choi A. (2009). Solid-state electrochemical gas sensors. Ionics.

[35] Chevallier L., Bartolomeo E.D., Grilli M.L., Briggs W.M.M., Wachsman E.D., Traversa E. (2008). Non-nernstian planar sensors based on YSZ with a Nb_2_O_5_ electrode. Sens. Actuators B Chem..

[36] Macam E.R., Blackburn B.M., Wachsman E.D. (2011). The effect of La_2_CuO_4_ sensing electrode thickness on a potentiometric NOx sensor response. Sens. Actuators B Chem..

[37] Di Bartolomeo E., Grilli M.L., Traversa E. (2004). Sensing mechanism of potentiometric gas sensors based on stabilized zirconia with oxide electrodes. J. Electrochem. Soc..

[38] Maurer B., Jacob E., Weisweiler W. (1999). Modellgasuntersuchungen mit NH_3_ und Harnstoff als Reduktionsmittel für die katalytische NO_x_-Reduktion. MTZ.

[39] Aris R. (1999). Elementary Chemical Reactor Analysis.

[40] Wijngarden R.J., Kronberg A., Westerterp K.R. (1998). Industrial Catalysis: Optimizing Catalysts and Processes.

[41] Topsoe N.Y. (1997). Catalysis for NO_x_ abatement-Selective catalytic reduction of NO_x_ by ammonia: Fundamental and industrial aspects. Cattech.

[42] Fujio Y., Plashnitsa V.V., Breedon M., Miura N. (2012). Construction of sensitive and selective zirconia-based CO sensors Using ZnCr_2_O_4_-based sensing electrodes. Langmuir.

[43] Lu G., Miura N., Yamazoe N. (1998). High-temperature NO or NO_2_ sensor using stabilized zirconia and tungsten oxide electrode. Ionics.

[44] Tronconi E., Nova I., Ciardelli C., Chatterjee D., Weibel M. (2007). Redox features in the catalytic mechanism of the “standard” and “fast” NH_3_-SCR of NO_x_ over a V-based catalyst investigated by dynamic methods. J. Catal..

[45] Ciardelli C., Nova I., Tronconi E., Konrad B., Chatterjee D., Ecke K., Weibel M. (2004). SCR for diesel engine exhaust aftertreatment: unsteady-state kinetic study and monolith reactor modelling. Chem. Eng. Sci..

[46] Grossale A., Nova I., Tronconi E. (2008). Study of a Fe-zeolite-based system as NH_3_-SCR catalyst for diesel exhaust aftertreatment. Catal. Today.

